# Self-association of Trimethylguanosine Synthase Tgs1 is required for efficient snRNA/snoRNA trimethylation and pre-rRNA processing

**DOI:** 10.1038/srep11282

**Published:** 2015-06-15

**Authors:** Kum-Loong Boon, Michael David Pearson, Martin Koš

**Affiliations:** 1 Biochemistry Center (BZH), University of Heidelberg, Im Neuenheimer Feld 328, 69120 Heidelberg, Germany; 2 Department of Cellular Biochemistry, Max Planck Institute for Biophysical Chemistry, Goettingen, Germany

## Abstract

Trimethylguanosine Synthase catalyses transfer of two methyl groups to the m^7^G cap of RNA polymerase II transcribed snRNAs, snoRNAs, and telomerase RNA TLC1 to form a 2,2,7-trimethylguanosine cap. While i*n vitro* studies indicate that Tgs1 functions as a monomer and the dimethylation of m^7^G caps is not a processive reaction, partially methylated sn(o)RNAs are typically not detected in living cells. Here we show that both yeast and human Tgs1p possess a conserved self-association property located at the N-terminus. A disruption of Tgs1 self-association led to a strong reduction of sn(o)RNA trimethylation as well as reduced nucleolar enrichment of Tgs1. Self-association of Tgs1p and its catalytic activity were also prerequisite to bypass the requirement for its accessory factor Swm2p for efficient pre-rRNA processing and snRNA trimethylation. The ability to self-associate might enable Tgs1 to efficiently dimethylate the caps of the targeted RNAs *in vivo*.

The m^7^G caps of RNA polymerase II transcribed snRNAs, snoRNAs, telomerase RNA TLC1 and selenoprotein mRNAs are tri-methylated by the methyltransferase Tgs1p through two successive methyl-transfer reactions from AdoMet to the N2 position of 7-methylguanosine^1^,^2^,^3^,^4^. The trimethylguanosine (TMG) cap modification is highly conserved throughout eukaryotes. However, it is not essential for the viability of the budding yeast *Saccharomyces cerevisiae*, and deletion of *TGS1* only causes a cold sensitive growth phenotype^1^. Although the steady state levels of snRNAs in *tgs1Δ* yeast are similar to that of wild type, deletion of *TGS1* results in genome-wide pre-mRNA splicing defects^5^, indicating a role for the TMG cap modification beyond simple snRNA/snoRNAs stabilisation. Indeed, the disruption of TGS1 in *D*. *melanogaster* leads to arrested pupal development^6^, and the disruption of TGS1 in mice leads to early embryonic lethality^7^, indicating an essential function of this protein during development.

The human Tgs1 (hTgs1) homolog was first designated as PIMT (PRIP-interacting protein with methyltransferase domain) for its interaction with PRIP (PPAR-interacting protein)^8^. The N-terminus of Tgs1 protein has no known domain, and varies in size across different species, while its C-terminus, which contains the methyltransferase domain, is more conserved^1^. Unlike yeast Tgs1p, the hTgs1p contains an additional large N-terminal domain. Interestingly, a truncated hTgs1p, consisting of amino acids (aa) 631–853 and thus missing the N-terminal domain, is able to rescue the yeast *tgs1Δ* cold sensitive phenotype and sn(o)RNA (snRNA, and snoRNA) trimethylation, demonstrating that the hTgs1p is truly the ortholog of yeast Tgs1p^9^. In contrast to hTgs1p, the yeast N-terminal domain of Tgs1p was found to be dispensable for binding to the common snRNP and snoRNP proteins, but essential for nucleolar localisation and snRNA trimethylation^10^.

The yeast Tgs1p has been shown by yeast two-hybrid, and *in vitro* interaction, to associate with Swm2 (synthetic with Mud2)^11^,^12^, a yeast protein with no known domains or homology to other proteins, and which does not seem to be conserved in humans. While *SWM2* is not essential, its deletion causes a similar genetic interaction profile, and nearly identical splicing defects as observed in *tgs1Δ*^5^,^12^. In yeast, Swm2p is localised to the nucleolus, and its nucleolar accumulation is dependent on the presence of Tgs1p^12^. Upon deletion of *SWM2*, Tgs1p selectively fails to trimethylate snRNAs, suggesting that Swm2p provides substrate specificity to Tgs1p toward this class of RNAs^12^. Both Tgs1p and Swm2p are also required for pre-rRNA processing, and deletion of either of these genes results in similar pre-rRNA processing defects^12^,^13^.

In this study, we show that Tgs1 contains a conserved self-association domain localised at its N-terminus, which is important for nucleolar accumulation and for efficient sn(o)RNA trimethylation in yeast. Disruption of Tgs1p self-association property does not interfere with the Tgs1p and Swm2p interaction. However, only overexpression of a self-association competent and catalytic active Tgs1p eliminates the pre-rRNA processing, and snRNA trimethylation defects seen in *swm2Δ* strains.

## Results

### Tgs1p self-interacts through its N-terminus

**Figure 1:**
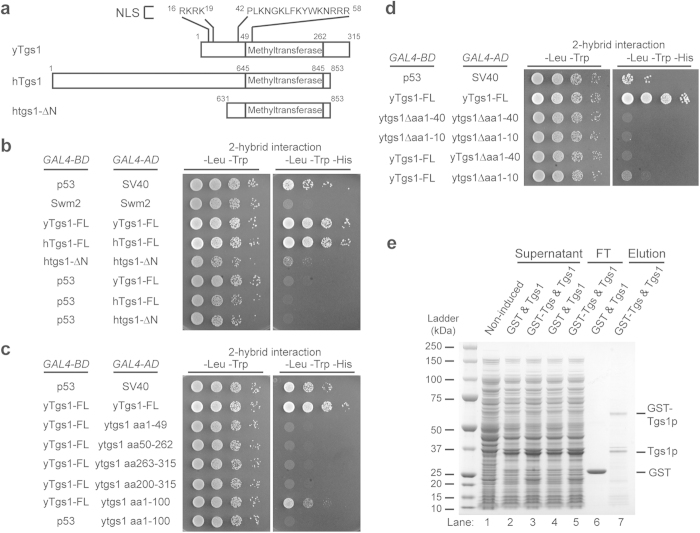
Tgs1p self-association region revealed by yeast two-hybrid analysis. **a**) Diagram showing the alignment of yeast and human Tgs1p. **b**) Yeast two-hybrid plasmids expressing Swm2p, hTgs1p, or yTgs1p were co-transformed into the PJ69-4A reporter strain and spotted in 10-fold serial dilutions on SDC-Trp-Leu, or SDC-Trp-Leu-His plates. Positive interactions allow growth on SDC-Trp-Leu-His plates. Yeast Tgs1p fragments (**c**), or truncated N-terminus Tgs1p (**d**) was tested for yeast two-hybrid interactions as described above. **e**) Recombinant proteins (GST and Tgs1p: lanes 2, 4, and 6, or GST-Tgs1p and Tgs1p: lanes 3, 5, and 7) were purified using Gluthathione sepharose beads and determined by mass spectrometry. FT: flow-through.

In our previous Tgs1p and Swm2p interaction studies we noticed indications that the yeast Tgs1 might self-interact. To confirm this observation, we tested the full-length Tgs1p self-association in the yeast two-hybrid assay. Tgs1p and Swm2p were fused to GAL4 DNA binding domain (GAL4-BD) to use as bait, or to GAL4 activation domain (GAL4-AD) to use as prey. Both bait and prey plasmids were then co-transformed into the PJ69-4A reporting yeast strain^14^, and the resulting yeast strains containing both plasmids were screened for Swm2p, or Tgs1p self-interaction. The combination of GAL4-BD fused to P53 and GAL4-AD fused to SV40 large-T antigen served as a positive control. The Swm2p showed no self-interaction *in vivo*, as the yeast strain containing both the bait and prey fusions of Swm2p failed to grow on –His selective plates (Fig. 1b). In contrast, the yeast strain expressing both the bait and prey fusions of yeast Tgs1p was able to grow on –His selective plates, suggesting that yeast Tgs1p self-interacts. To determine if this self-association is a conserved property of Tgs1p, we next tested the human homolog of Tgs1p by yeast two-hybrid analysis as mentioned above. Indeed, the human full-length Tgs1p also showed self-interaction and was able to grow on –His selective plates (Fig. 1a,b). We next tested the hTgs1p truncation protein (aa631-853) that was previously shown to rescue the yeast *TGS1* deletion^9^. To our surprise, with its N-terminus truncated, the self-association of human Tgs1 is severely reduced (Fig. 1b). As negative control, a GAL4-BD-p53 and GAL4-AD-Tgs1 expressing strain failed to grow on –His selective plates, suggesting that the Tgs1p self-interaction result was not a false positive (Fig. 1b). It is also unlikely that the observed results are due to autoactivation, as full-length GAL4-BD-yTgs1 fails to grow on –His plates in the absence of full-length GAL4-AD-Tgs1 (Fig. 1c,d).

As our lab uses budding yeast as a research model, we further focused on the region that is responsible for yeast Tgs1p self-interaction. We utilised our previously published Tgs1p yeast two-hybrid constructs^12^, where the full-length Tgs1p is fused to GAL4-BD to use as bait, while the Tgs1p MTase domain (aa50-262)^10^ and fragments between the MTase domain were fused to the GAL4-AD and used as prey (Fig. 1a,c). The full-length yTgs1p shows self-interaction, but not the fragments of Tgs1p (aa1-49, aa50-262, or aa263-315). It is worth noting, that the methyltransferase domain (aa50-262) alone is not capable of self-association (Fig. 1a,c). These results suggest that full-length protein is required for the self-association. Alternatively, the interaction site could lie between the Tgs1 fragments, with the break points of aa49, or aa262 disrupting the interaction. To examine this further, we tested the Tgs1 fragments composed of aa1-100, and aa200-315 for yeast two-hybrid interactions. Full-length Tgs1p showed a positive interaction with the 100 N-terminal amino acids (yTgs1aa1-100), but not with the C-terminus fragment (aa200-315), confirming that the intact N-terminus (aa1-100) is sufficient for the yTgs1 self-interaction (Fig. 1c).

We then tested the self-association of Tgs1p lacking different sections of the N-terminus. Tgs1p truncated without the first 40 amino acids (aa1-40) failed to activate the His reporter and showed no growth on –His selective plates (Fig. 1d). However, there is a proposed bipartite nuclear localisation signal (NLS) at positions aa16-19 and aa42–58^10^ (Fig. 1a), which would have been affected by this deletion. Therefore, we tested a smaller deletion that preserves the intact NLS. Interestingly, deletion of only the first 10 amino acids was sufficient to abolish fully Tgs1p self-association (Fig. 1d). This again suggested that the Tgs1p self-interaction required an intact Tgs1 aa1-100 protein fragment. The combination of the full-length Tgs1p and the Tgs1p aa1-10 truncation showed very slight growth above background on –His plates suggesting a weak interaction (Fig. 1d). Interestingly, the N-terminus lacking mutant hTgs1aa631-853 (htgs1-ΔN, and Fig. 1b) also showed severe reduction of growth when compared to full-length human Tgs1p suggesting that self-association through the N-terminus is conserved between human and yeast (Fig. 1b–d).

The Tgs1p yeast two-hybrid *in vivo* interaction could be either direct or indirect that influence by other cellular proteins. To determine whether Tgs1p can interact with itself in the absence of other yeast proteins, we co-expressed GST-Tgs1p using a pRFSDuet-1 vector, and Tgs1p using a pETDuet-1 vector in *E*. *coli*. The untagged Tgs1p could be co-purified with GST-Tgs1p (Fig. 1e, lane 7), but not with GST alone (Fig. 1e, lane 6) confirming that Tgs1p can indeed self-interact in the absence of other yeast proteins.

### Tgs1p self-association is required for nucleolar localisation and efficient sn(o)RNA trimethylation

**Figure 2:**
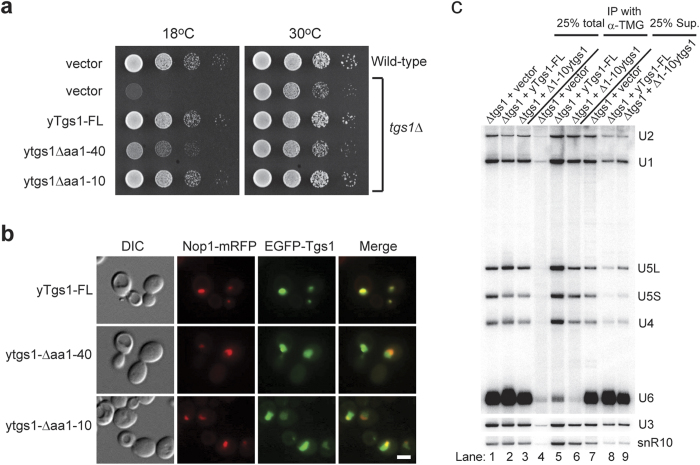
The N-terminus of yTgs1p is required for efficient nucleolar localisation and sn(o)RNA trimethylation. **a**) Yeast *tgs1Δ* cells transformed with vector, yTgs1-FL, ytgs1Δaa1-40, or ytgs1Δaa1-10 were serially diluted, and grown on –Ura plates. **b**) The subcellular localisation of Tgs1p was analysed for co-localisation with nucleolar marker Nop1-mRFP. DIC: Differential interference contrast. Scale bar, 2 μm. **c**) Total RNA from ytgs1Δaa1-10 was immunoprecipitated with anti-TMG antibodies and analysed by Northern blotting.

To understand the importance of Tgs1p self-interaction, we tested Tgs1p N-terminus truncation mutants for complementation of the cold-sensitive phenotype of *tgs1Δ*. Interestingly, although both the Δaa1-40 and Δaa1-10 truncated versions of Tgs1p lost their self-interaction capability, they rescue the *tgs1Δ* cold-sensitive phenotype to different degrees. While the Δaa1-10 Tgs1 complements as well as full-length Tgs1p, the Δaa1-40 Tgs1p shows slow growth at 18 °C. At 30 °C, all yeast strains tested show similar growth and colony size (Fig. 2a).

To analyse the effect of various N-terminal deletions on subcellular localisation of Tgs1p we utilised an EGFP-Tgs1 fusion protein. Wild-type Tgs1p fused with EGFP at its N-terminus was shown to be functional and complemented the *TGS1* deletion^1^,^10^,^12^. Sub-cellular localisation studies in yeast show that Tgs1p is localised to the nucleolus, and the N-terminus of Tgs1p was previously proposed to contain a bipartite classical SV40 nuclear localisation signal (NLS) (aa16-19, and aa42-58)^10^. We next tested the localisation of an N-terminal truncation mutant of Tgs1p that contains the NLS, but lacks its self-association property. To indicate the nucleolar compartment, each yeast strain was co-transformed with a plasmid expressing the nucleolar protein Nop1 protein fused to mRFP at its C-terminus. In agreement with previous reports^10^,^12^, full-length EGFP-Tgs1p was found in the nucleolus and co-localised with the nucleolar marker Nop1 (Fig. 2b, upper panel). The truncation Tgs1Δaa1-40, in which the NLS is partially disrupted, was not as efficiently localised to nucleolus and showed mild cytoplasmic staining, however it remained enriched in the nucleus (Fig. 2b, middle panel). Interestingly, Tgs1Δaa1-10, with an intact bipartite NLS, also showed reduced colocalisation with Nop1 and increased staining of nucleoplasm (Fig. 2b, lower panel). This suggests that the self-association property of Tgs1p *per se* is required for the efficient nucleolar concentration of Tgs1p. It is worthy to note that the Δaa1-40, possessing only one part of the proposed bipartite NLS, remained enriched in the nucleus, indicating that a sufficient nuclear localisation signal is present in the remaining part of Tgs1, consistent with the previous report in Mouaikel *et al.*, 2003^10^.

As Tgs1Δaa1-10 could rescue the cold sensitive phenotype of *tgs1Δ,* we next investigated the sn(o)RNA trimethylation efficiency in this mutant. RNAs containing a trimethylated cap were immunoprecipitated directly from yeast total RNA by using R1131 antibodies, which specifically recognises the m_3_G cap structure^15^, followed by Northern blotting analysis. Total RNA was used instead of yeast cell extract in the immunoprecipitation to eliminate the presence of cellular protein that could interfere with the binding of the RNA cap structure by the R1131 antibodies. As seen in Fig. 2c, trimethylated sn(o)RNAs were efficiently immunoprecipitated from yeast containing full-length Tgs1p. Interestingly, although Tgs1Δaa1-10 rescued the cold sensitive phenotype of *tgs1Δ* as well as full-length Tgs1p (Fig. 2a), the amount of immunoprecipitated snRNAs and snoRNAs were clearly reduced to about half of the wild type level (Fig. 2c). This observation indicates that self-association of Tgs1p is required for efficient sn(o)RNA trimethylation.

### Tgs1p self-association and its catalytic activity are required to bypass lack of Swm2p in RNA processing

**Figure 3:**
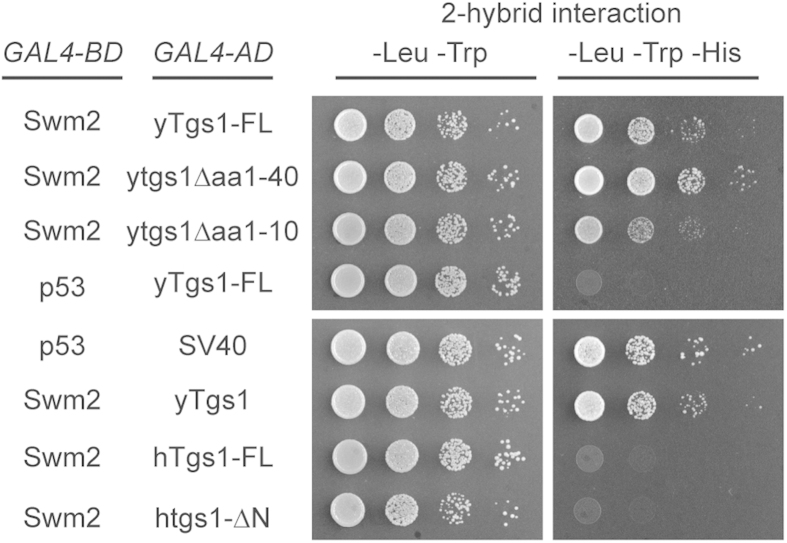
Yeast two-hybrid analysis of interactions between Swm2p and yeast or human Tgs1p.

The Tgs1p self-association domain is located at its N-terminus (Fig. 1a–d). Interestingly, our previous work showed that the N-terminus of Tgs1p (aa1-100) also interacts with Swm2p^12^. To test the effect of N-terminal truncations of Tgs1p, which lose self-association capability, on the interaction with Swm2p, we again employed the yeast two-hybrid analysis. We fused Swm2p to GAL4-BD and Tgs1p (full-length, Δaa1-10, or Δaa1-40) to GAL4-AD, and the constructs were co-transformed into the PJ69-4A yeast strain, and tested for growth on –His plates (Fig. 3). Surprisingly, although truncated Tgs1 lost its self-association property, its interaction with Swm2p remained unaltered (Fig. 3). We next tested the potential interaction between yeast Swm2p and human Tgs1p by yeast-hybrid analysis. The GAL4-BD-Swm2, and GAL4-AD-hTGS1 were co-transformed into the PJ69-4A yeast strain. In contrast to yTgs1p, we found no interaction of Swm2p with either full-length or N-terminus truncated human Tgs1p (Fig. 3), as the reporter yeast cells failed to grow on –His plates, suggesting that the interaction of Swm2p and Tgs1p is yeast specific.

**Figure 4:**
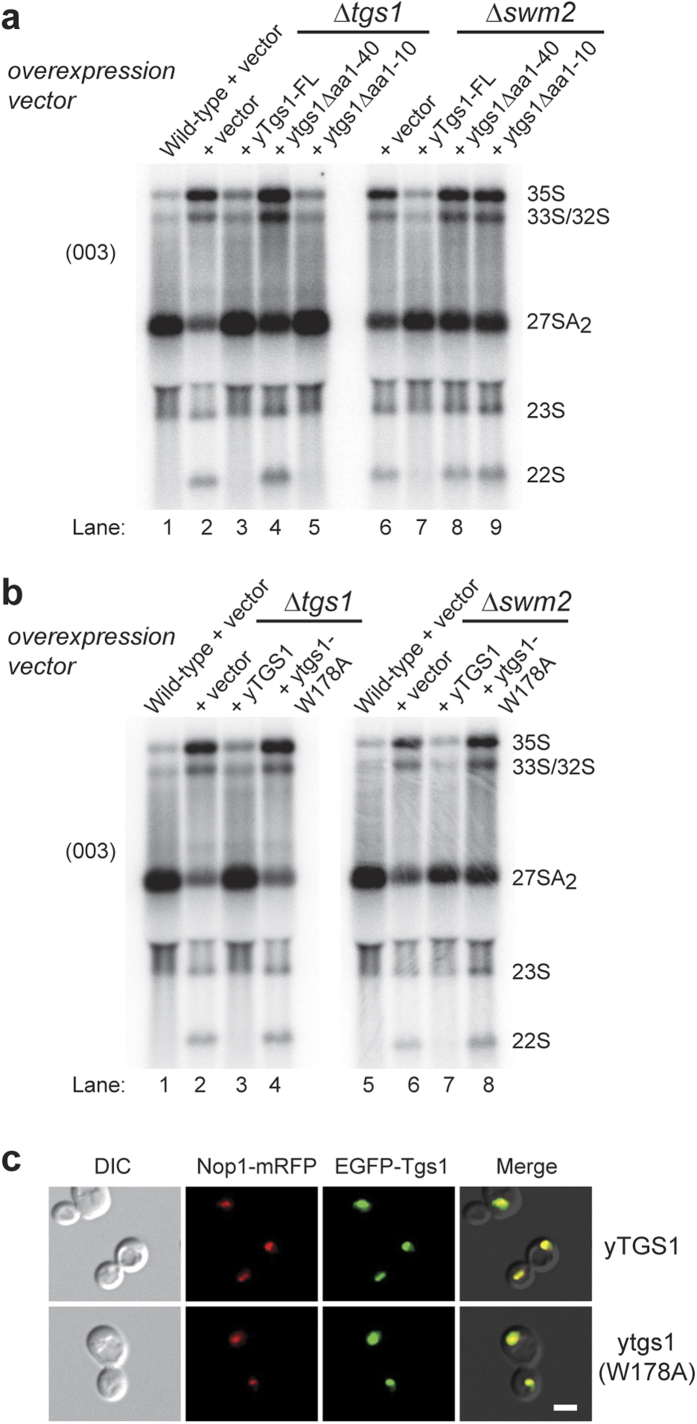
Analysis of pre-rRNA processing defects in strains overexpressing full-length and truncated Tgs1p. **a**-**b**) Total RNA from wild-type, *tgs1Δ*, or *swm2Δ* yeast strains transformed with either an empty PRS316-GFP vector or a vector expressing yTgs1-FL, ytgs1Δaa1-40, ytgs1Δaa1-10, or *ytgs1-W178A* was extracted and analysed by Northern blotting with a probe 003 complementary to ITS1 region between cleavage points A2 and A3 of yeast pre-rRNA. **c**) yTgs1-FL, or *ytgs1-W178A* are colocalised with nucleolar marker Nop1-mRFP. DIC: Differential interference contrast. Scale bar, 2 μm.

Yeast strains with a deletion of *TGS1*, or *SWM2,* display a cold-sensitive phenotype, with pre-rRNA processing defects leading to accumulation of 35S and 22S pre-rRNAs, and a reduction in 27SA_2_^12^. We next tested the effect of ectopic overexpression of full-length and truncated Tgs1p on the pre-rRNA processing in both *tgs1Δ*, and *swm2Δ* yeast strains. The strains were transformed with a vector containing a *PGK1* promoter driving expression of Tgs1, or an empty vector. Overexpression of the full-length Tgs1p eliminated pre-rRNA processing defects in either *tgs1Δ* or *swm2Δ* (Fig. 4a, lane 3, and 7). Interestingly, the complementation with self-association defective Tgs1p mutants provided a different outcome. In *tgs1Δ*, Tgs1Δaa1-10 complemented the pre-rRNA processing as efficiently as full-length Tgs1p (Fig. 4a, lane 5). On the other hand, Tgs1Δaa1-40, which has a further N-terminal truncation, showed an increased amount of 27SA_2_, but the 35S and 22S aberrant precursors remained accumulated (Fig. 4a, lane 4). In the case of *swm2Δ*, the overexpression of both self-association defective Tgs1p mutants (Tgs1Δaa1-10, or Tgs1Δaa1-40) improved only the level of 27SA_2_ transcripts, but the 35S and 22S aberrant precursors remained accumulated (Fig. 4a, lane 8–9), which suggests that self-association of Tgs1p is important for pre-rRNA processing in the absence of Swm2 protein.

As it has been reported that the catalytic function of yTgs1 is not required for rRNA processing in yeast^13^, we next tested whether this holds true also in the absence of Swm2p. Surprisingly, the catalytically inactive mutant *ytgs1-W178A* did not rescue the rRNA processing in neither *swm2Δ* nor *tgs1Δ* (Fig. 4b, lane 4 and 8). In both strains, processing of the primary transcript 35S pre-rRNA and 22S precursors were readily detectable, where the 27SA_2_ transcript remain reduced (Fig. 4b, lane 4 & 8), in a pattern identical to the *tgs1Δ* defects. The subcellular localisation of *tgs1-W178A* was not changed (Fig. 4c)^1^, remaining enriched in the nucleolus and co-localising with Nop1-mRFP.

### Tgs1p self-association and its catalytic activity are required to bypass lack of Swm2p in snRNAs trimethylation

**Figure 5:**
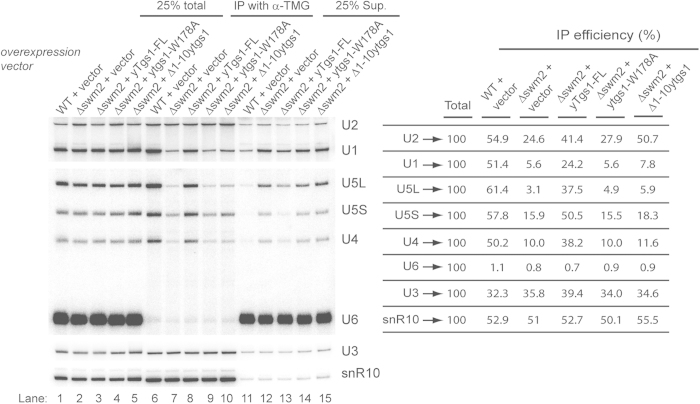
The self-association competent and catalytically active Tgs1p is required to trimethylate snRNAs in the absence of Swm2p. Total RNA from wild-type, or *swm2Δ* yeast strains transformed with either an empty PRS316-GFP vector, or a vector expressing yTgs1-FL or ytgs1Δaa1-10 was immunoprecipitated with anti-TMG antibodies and analysed by Northern blotting. Quantification of the immunoprecipitation efficiency as a fraction of input is presented in the table on the left. The total RNA input was set as 100%. Sup: supernatant.

Our previous results indicated that with the deletion of *SWM2*, the trimethylation of snRNAs, but not snoRNAs^12^, is impaired (Fig. 5). To analyse the snRNA trimethylation efficiency in *swm2Δ* yeast strains with or without additional Tgs1p, we immunoprecipitated the total RNA prepared from these yeast strains using anti-TMG antibodies, and then analysed the RNAs by Northern blotting (Fig. 5). Consistent with the results from pre-rRNA processing, expression of additional full-length Tgs1p in the *swm2Δ* strain improved overall trimethylation of snRNA when compared to an empty vector (Fig. 5, lanes 7-8). On the other hand, the catalytically inactive *ytgs1-W178A* and self-association defective Tgs1p failed to improve the efficiency of the majority of snRNA cap trimethylation when compared to full-length wild type Tgs1p (Fig. 5, lane 8–10). Unexpectedly, Tgs1Δaa1-10 selectively improved the trimethylation of only U2 snRNA, but not the other snRNAs tested (Fig. 5, lane 10). The immunoprecipitation of U3 and snR10 snoRNAs showed similar levels in all tested yeast strains (Fig. 5, lane 6–10). These results show that an excess of self-association competent and catalytic active Tgs1p is a prerequisite to bypass the requirement of Swm2p for snRNA trimethylation.

## Discussion

Here we show that yeast and human Tgs1 proteins possess a conserved self-association domain at their N-terminus. Although the N-terminus truncation Tgs1p mutants were stably expressed^10^, they failed to be enriched in the nucleolus, and also failed to trimethylate sn(o)RNAs. This could be due to disruption of the bipartite NLS present at its N-terminus, which was suggested to be required for the proper nucleolar localisation of Tgs1p^10^. However, the Tgs1Δaa1-10 mutant, which has an intact NLS, but has lost its self-association capacity, was also not able to efficiently trimethylate sn(o)RNAs. In addition, self-association defective Tgs1 mutants are clearly localised in the nucleus, but fail to be enriched in the nucleolus. This suggests that the bipartite NLS alone might not be sufficient for Tgs1p nucleolar localisation and that the Tgs1 self-association property is also required. The truncated Tgs1Δaa1-10 is also deficient in trimethylation of both snRNA and snoRNA, which suggests that the defect is general and not restricted to a singular class of RNAs. This indicates that a functional self-association between Tgs1p proteins is required for efficient sn(o)RNA trimethylation.

The results of copurification of differently tagged Tgs1 co-expressed in *E*. *coli* in the absence of other yeast proteins suggests that Tgs1 alone can indeed form a larger complex. However, we cannot formally conclude whether Tgs1p is in the form of a dimer, or higher order oligomers. Unfortunately, our attempts to purify full-length and N-terminally truncated Tgs1 in a quality suitable for MALS were unsuccessful.

In the absence of Swm2p, a Tgs1p interacting protein, yeast cells display pre-rRNA processing defects, as well as a loss of snRNA trimethylation. Our previous work showed that Swm2p interacts with Tgs1p at its N-terminus, from aa1-100^12^. In this study, we showed that deletion of aa1-40 of Tgs1 does not interrupt the Swm2 interaction, and thus the minimum Tgs1p interaction region can be narrowed down to aa41-100. Interestingly, in this study we found that the observed requirement of Swm2p in RNA processing can be bypassed by overexpression of wild type Tgs1p but not self-association defective mutants that are still capable in Swm2p interaction.

We have also observed that catalytically inactive *ytgs1-W178A* could not rescue pre-rRNA processing defects in the *Δtgs1* strain in our experiments, contrary to previous report of Colau *et al.* 2004^13^. Whether these discrepancies arise from the usage of parental strains with different genetic backgrounds is unclear. Another possible explanation is the difference in the temperatures used during the experiment. While we permanently cultured all strains at semi-permisive temperature of 23 °C, Colau *et al.* used cultures shifted for 6–9 hours to non-permisive 16 °C, a temperature, at which yeast strains lacking Tgs1 fail to grow^1^,^13^. The relative levels of various pre-rRNA intermediates are likely to differ between semi-permisive and non-permisive temperatures. Our results suggest that the major cause of pre-rRNA processing defects at the semi-permisive temperature is a defect in sn(o)RNA trimethylation and that the Tgs1 catalytic activity, and not only the protein *per se*, is required for correct pre-rRNA processing during ribosome biogenesis.

Lack of either Tgs1p or Swm2p leads to splicing and pre-rRNA processing defects. While we cannot formally exclude that both Tgs1p and Swm2p have independent roles in pre-rRNA processing, their role is likely mutually dependent and indirect. A simple model consistent with observations presented here and in previous reports is the following. The lack of Tgs1p or its catalytic activity leads to undermethylation of snRNAs and snoRNAs affecting splicing and rRNA modifications. The more critical are probably the splicing defects, reducing splicing of mRNAs for ribosomal proteins and splicing of the U3 snoRNA precursor – both playing direct role in ribosome biogenesis and pre-rRNA processing. The self-association of Tgs1 is not absolutely essential, but greatly enhances the efficiency of sn(o)RNAs methylation (Fig. 2c, lanes 5 and 6). Swm2p aids Tgs1p to methylate efficiently snRNAs, but again it is not essential. In the absence of Swm2, overexpression of self-association capable wild type Tgs1 provides reduced but sufficient level of snRNA trimethylation, rescuing the pre-rRNA processing. In *tgs1Δ,* the overexpression of Tgs1-∆aa1-10 compensates for its reduced activity. However, the combined lack of Swm2 together with the strongly diminished activity of the self-association deficient Tgs1-∆aa1-10, is not able to sustain required levels of snRNA methylation, resulting in splicing and consequently also pre-rRNA processing defects.

The fact that Tgs1p can self-associate suggests a possible mechanism of sn(o)RNA methylation *in vivo*. The m^7^G cap dimethylation by Tgs1 requires two successive methyl-transfer reactions from AdoMet to the N2 position of 7-methylguanosine. Based on *in vitro* experiments, it was suggested that Tgs1 acts as a monomer, that cap dimethylation is not a processive reaction, and that Tgs1 dissociates from the substrate after the first methylation^3^. However, these experiments used truncated Tgs1, which lacked the N-terminus and was therefore incapable of self-association. It is tempting to speculate, that in the Tgs1 multimer (perhaps a homodimer), each Tgs1 monomer transfers in succession one of the methyl groups. This would allow efficient dimethylation of the RNA caps without the need to release the substrate sn(o)RNA. The inability of the *ytgs1-W178A* catalytic mutant to complement the snRNA trimethylation defect in *swm2Δ* (i.e. in the presence of wild type endogenous Tgs1p) suggests that each of the self-associated Tgs1 proteins must be catalytically active. Accordingly, in strains with self-association deficient Tgs1, the levels of trimethylated sn(o)RNAs would be reduced, while over-expression of Tgs1 would increase and compensate the levels of trimethylation, which is in agreement with our observations.

In summary, we show that catalytically active and self-association competent Tgs1p is required for efficient trimethylation of sn(o)RNAs in yeast. Interestingly, the human Tgs1 also possesses self-association capacity through its N-terminus. These results suggest that Tgs1p self-association might be part of a conserved mechanism by which Tgs1 protein dimethylates its target RNAs.

## Material and Methods

### Yeast strains and plasmids

The *S. cerevisiae* strains and plasmids used in this study are listed in Table 1, and the oligonucleotides used are listed in Table 2. Yeast transformation procedure was performed as described in Gietz *et al.* 2002^16^.

### RNA extraction, immunoprecipitation and Northern analysis of RNA

RNA was extracted as previously described in Tollervey and Mattaj, 1987^17^. Yeast strains containing PRS316 derivative plasmids (empty vector or with PGK1 promoter controlled wild type or mutant *TGS1*) were grown in –Ura dropout broth at 23 °C before being harvested. 10 OD of yeast cells in log phase (OD_600_ 0.3–0.5) were pelleted, mixed with 0.3 ml of GTC mix (4 M Guanidine Thiocyanate, 0.05 M Tris pH8, 0.01 M EDTA pH8, 2% Sarkosyl, 1% β mercaptoethanol), then vortexed with zirconia beads for 10 mins before being heated at 65 °C for 5 mins. Subsequently, 0.15 ml of NaAc mix (0.1 M sodium acetate, 1 mM EDTA, 1 mM Tris-HCl, pH 8.0) was added, and the solution was extracted twice with phenol/chloroform/iso-amylalcohol. The total RNA was then precipitated by ethanol. Immunoprecipitation was performed as described in Hausmann *et al.*, 2007^18^ in IPP150 buffer (6 mM HEPES (pH7.9), 150 mM NaCl, 5 mM MgCl_2_, 0.1% Nonidet P-40) with protein A-Sepharose (GE Healthcare) bound anti-TMG (R1131) antibodies^15^. For immunoprecipitation, total RNA was incubated with protein A-Sepharose bound anti-TMG antibodies sepharose beads on a rotating wheel for 2 hour at 4 °C. The beads bound RNA was digested with proteinase K solution (50 mM Tris-HCl (pH7.5), 5 mM EDTA, 1.4% SDS, proteinase K (2 μg/μl)) for 30 minutes at 37 °C. Then the RNA was extracted twice with phenol-chloroform, concentrated by ethanol precipitation, and fractionated on a 6% (wt/vol) polyacrylamide-8 M urea gel, or 1.2% glyoxal agarose gel. The sn(o)RNAs, and rRNAs were detected by Northern analysis with end-labeled deoxyoligonucleotides complementary to the targeted sn(o)RNAs (Table 2). The amount of radioactivity in RNA bands derived from a representative pulldown was visualised by Fuji imager system and quantified by AIDA software.

### Yeast two-hybrid analysis

For yeast two-hybrid analysis, the bait plasmids with fusions to the GAL4 DNA binding domain, and prey plasmid with fusions to GAL4 activation domain (G4AD), were cotransformed into the reported strain PJ69-4A^14^. Yeast two-hybrid interaction were performed by spotting the transformants in a 10-fold serial dilution on synthetic dextrose complete (SDC) media SDC-Trp-Leu, or SDC-Trp-Leu-His (*HIS3* reporter), and grown at 30 °C. As a positive control, the combination of plasmids pVA3-1, and pTD1-1 (Clontech Laboratories, Inc) were used. These plasmids also served as negative control when co-transformed with plasmids under investigation. PJ69-4A strains with a positive yeast two-hybrid interaction are able to survive on the SDC-Trp-Leu-His media.

### Protein extracts preparation

For recombinant protein production, *Escherichia coli* BL21 (DE3) cells (Novagen) grown overnight at 37 °C in 25 ml LB broth containing 100 μg/ml ampicillin, and 25 μg/ml kanamycin were inoculated into 500 ml LB broth, and grown at 37 °C. At OD600_nm_ of 0.8, IPTG was added to concentration of 0.75 mM, and the culture was further incubated for 3 h at 30 °C. Cells were pelleted, resuspended in 25 ml lysis buffer (150 mM NaCl, 50 mM Tris-HCl (pH 7.4)), and suspended cells were lysed by micro-fluidiser. The cell lysates were then spun at 15,500 rpm for 20 min, at 4 °C. The cleared supernate, containing both GST-tagged recombinant proteins, was removed and subsequently purified by standard Glutathione Sepharose purification method. Purified proteins were fractionated on a Novex NuPAGE 4–12% bis-Tris gel (Invitrogen) and stained with colloidal Coomassie (Roth).

### Live cell imaging

Cells were grown in SDC-Leu-Ura liquid medium in log phase at 30 °C. Fluorescence microscopy was performed using Imager Z1 (Carl Zeiss). Pictures were acquired with AxioCamMRm camera (Carl Zeiss) and software Axio Vision 4.3 (Carl Zeiss). Pictures were then exported as jpeg files and processed in Adobe PhotoShop CS6.

## Additional Information

**How to cite this article**: Boon, K.-L. *et al.* Self-association of Trimethylguanosine Synthase Tgs1 is required for efficient snRNA/snoRNA trimethylation and pre-rRNA processing. *Sci. Rep.* **5**, 11282; doi: 10.1038/srep11282 (2015).

## Figures and Tables

**Table 1:** Plasmids and yeast strains used in this study.

Plasmid or strain	Purpose or genotype	Source or Kos lab reference
Plasmids		
PRS316-GFP	*CEN, URA3, P*_*PGK1*_*-EGFP*	12
EGFP-yTGS1	*CEN-URA3-P*_*PGK1*_*-EGFP-yTGS1*	12
EGFP-yTGS1-aa10-315	*CEN, URA3, P*_*PGK1*_*-EGFP-yTgs1 (aa10-315)*	This study, pMK452
EGFP-yTgs1-aa40-315	*CEN, URA3, P*_*PGK1*_*-EGFP-yTgs1 (aa40-315)*	This study, pMK453
EGFP-yTgs1-W178A	*CEN, URA3, P*_*PGK1*_*-EGFP-yTgs1 (W178A)*	This study, pMK454
NOP1-mRFP	*CEN-URA3-P*_*NOP1*_*-NOP1-mRFP*	19
pGADT7	2 μ, *Leu2*, G4AD-HA	Clontech Laboratories, Inc.
pGBKT7	2 μ, *TRP1*, G4BD-c-myc	Clontech Laboratories, Inc.
pVA3-1	aa 72-390 of murine p53 expressed from pAS1_*CYH2*_; 2 μ, *TRP1*, *pADH1*, N-terminal G4BD	Clontech Laboratories, Inc.
pTD1-1	aa 87-708 of SV40 large T antigen expressed from pACT2; 2 μ, *Leu2*, *pADH1*, N-terminal G4AD	Clontech Laboratories, Inc.
pGAD-yTgs1-FL	pGADT7-based for production of yTgs1p (aa1-315)	12
pGAD-yTgs1-aa50-262	pGADT7-based for production of yTgs1p (aa50-262)	12
pGAD-yTgs1-aa263-315	pGADT7-based for production of yTgs1p (aa263-315)	12
pGAD-yTgs1-aa1-49	pGADT7-based for production of yTgs1p (aa1-49)	12
pGAD-yTgs1-aa200-315	pGADT7-based for production of yTgs1p (aa200-315)	12
pGAD-yTgs1-aa1-100	pGADT7-based for production of yTgs1p (aa1-100)	12
pGAD-yTgs1-aa10-315	pGADT7-based for production of yTgs1p (aa10-315)	This study, pMK457
pGAD-yTgs1-aa40-315	pGADT7-based for production of yTgs1p (aa40-315)	This study, pMK458
pGAD-hTgs1-FL	pGADT7-based for production of Tgs1p (aa1-853)	This study, pMK459
pGAD-hTgs1-aa631-853	pGADT7-based for production of Tgs1p (aa631-853)	This study, pMK460
pGAD-SWM2	pGADT7-based for production of Swm2p	This study, pMK463
pGBK-yTgs1-FL	pGBKT7-based for production of yTgs1p (aa1-315)	This study, pMK464
pGBK-yTgs1-aa10-315	pGBKT7-based for production of yTgs1p (aa10-315)	This study, pMK465
pGBK-yTgs1-aa40-315	pGBKT7-based for production of yTgs1p (aa40-315)	This study, pMK466
pGBK-hTgs1-FL	pGBKT7-based for production of hTgs1p (aa1-853)	This study, pMK467
pGBK-hTgs1-aa631-853	pGBKT7-based for production of hTgs1p (aa631-853)	This study, pMK468
pGBK-SWM2	pGBKT7-based for production of Swm2p	12
pETDuet-1	Expression vector; ColE1 replicon, T7 promoters, Ap^r^	Novagen
pETDuet-yTGS1	pETDuet-1-based for production of 6His-yTgs1p	This study, pMK496
pRSFDuet-1	Expression vector; RSF replicon, T7 promoters, Kn^r^	Novagen
pRSFDuet-GST	pRFSDuet-1-based for production of 6His-GST	This study, pMK497
pRSFDuet-GST-yTgs1	pRFSDuet-1-based for production of 6His-GST-yTgs1	This study, pMK498
Yeast strains		
BY4741	MATa*, his∆1, leu2∆0, met15∆0, Ura3∆0*	Euroscarf
*tgs1Δ*	*tgs1::kanMX6*, otherwise as BY4741	12
*swm2Δ*	*swm2::His3MX6*, otherwise as BY4741	12
Y2H strain PJ69-4A	*MAT*a, trp1-901, leu2-3,112, ura3-52, his3-200, gal4, gal80, GAL2-ADE2, *LYS2*::GAL1-HIS3, *met2*::GAL7-lacz	14

**Table 2:** Deoxyoligonucleotides used in this study.

Name	Probe	Source or Kos lab reference
	Northern blotting	
U1	CACGCCTTCCGCGCCGT	20
U2	CTACACTTGATCTAAGCCAAAAG	20
U4	AGGTATTCCAAAAATTCCC	20
U5	AAGTTCCAAAAAATATGGCAAGC	20
U6	ATCTCTGTATTGTTTCAAATTGACCAA	20
003	TGTTACCTCTGGGCCC	21
snR10	GCTGTTAAATTTGCGTT	21
	Oligonucleotides for generating plasmid constructs	
hTGS1-Spe1-F-004	GGACTAGTTGCTGCGAGAAGTGGAGCCGCGT	This study, oMK758
hTGS1-Xma1-R-2562	TCCCCCCGGGTTAGGTTTCAGAGGCTGGTCTTC	This study, oMK757
hTgs1-Sfi1-F-004	CATATGGCCATGGAGGCCGAATGCTGCGAGAAGTGGAGCCGCGT	This study, oMK838
hTGS1-Nde1-F-1891	GGAATTCCATATGGTGAATGGTCTGCCTCCT	This study
hTGS1-Xho1-R-2562	CCGCTCGAGTTAGGTTTCAGAGGCTGGTCTT	This study, oMK860
yTGS1-Nde1-F-121	GGAATTCCATATGTCAAAACCGTTGAAAAATGGT	This study, oMK844
yTgs1-Xma1-F-121	TCCCCCCGGGTCAAAACCGTTGAAAAATGGT	This study, oMK849
yTGS1-Nde1-F-31	GGAATTCCATATGATAAAACATGCGGCACGCAAAAG	This study, oMK856
yTGS1-Xma1-F-31	TCCCCCCGGGATAAAACATGCGGCACGCAAAAG	This study, oMK855
yTGS1-Xma1-R-968	TCCCCCCGGGGATTCAACTTGAACAAGTGTTTAACC	This study, oMK444
yTgs1-HindIII-R-968	GGGAAGCTTGATTCAACTTGAACAAGTGTTTAACC	This study, oMK510
yTgs1-Not1-R-968	AAGGAAAAAAGCGGCCGCGATTCAACTTGAACAAGTGTTTAACC	This study, oMK706
yTGS1-Xma1-F-004	TCCCCCCGGGAGGACATTTATTCATGCTTCG	This study, oMK840
yTGS1-HindIII-R-147	GGGAAGCTTAAGTTTACCATTTTTCAACGGT	This study, oMK841
yTGS1-W178A-R	TTTCTTAAGTATTCTGGACCACCGGCCGGGGGTGATCCAAATACACAGTCGTACTT	This study, oMK779
YNR004W-Xma1-F-004	TCCCCCCGGGTATAGATTTATACAATTACTC	This study, oMK787
YNR004W-Xho1-R-441	CCGCTCGAGTTATTCTTGTATGAAATCATTC	This study, oMK788
GST-Pst1-F-004	AACTGCAGTCCCCTATACTAGGTTATT	This study, oMK545
GST-Not1-R-660	AAGGAAAAAAGCGGCCGCTCAATCCGATTTTGGAGGATGGT	This study, oMK546
